# Supplementary material for: Using Machine Learning Algorithms to Develop a Clinical Decision-Making Tool for COVID-19 Inpatients
Source: Int J Environ Res Public Health. 2021 Jun 9;18(12):6228. doi: 10.3390/ijerph18126228 (PMC8296041; doi:10.3390/ijerph18126228)
Supplement: Supplementary file 1 [file ijerph-18-06228-s001.zip › ijerph-1207572-supplementary.pdf]

Supplementary Tables and Figures

Table S1: List and definitions of independent predictor variables

Independent Predictor Variables (p=44)	Definition
Age	The number of completed years since self-reported date of birth (years)
Gender	Self-reported gender by study participant (male of female)
Ethnicity	Self-reported ethnicity by study participant (Caucasian or BAME). 'Mixed' ethnicities and 'other' ethnicities were classified as BAME.
Oxygen Saturations	The lowest oxygen saturation reading obtained by nursing staff on the day of COVID-19 clinical presentation whilst an inpatient (%)
Respiratory Rate	The highest respiratory rate reading obtained by nursing staff on the day of COVID-19 clinical presentation whilst an inpatient (breaths per minute)
Temperature	The highest temperature reading obtained by nursing staff on the day of COVID-19 clinical presentation whilst an inpatient (°Celsius to 1 decimal point)
Obesity	A body mass index more than 30, or a clinical diagnosis of obesity documented within the patients past medical history (Yes or No)
Previous Venous Thromboembolism	A confirmed clinical diagnosis of any venous thromboembolism documented within the patients past medical history or a patient who is prescribed a treatment dose of anticoagulation (Yes or No)
Chronic Obstructive Pulmonary Disease	A confirmed clinical diagnosis of emphysema, bronchitis, or chronic obstructive pulmonary disease documented within the patients past medical history, or, a study participant with smoking history who is prescribed a long-term bronchodilator or corticosteroid inhaler (Yes or No)
Bronchiectasis	A confirmed clinical diagnosis of bronchiectasis documented within the patients past medical history (Yes or No)
Asthma	A confirmed clinical diagnosis of asthma documented within the patients past medical history (Yes or No)
Interstitial Lung Disease	A confirmed clinical diagnosis of interstitial lung disease or pulmonary fibrosis documented within the patients past medical history (Yes or No)
Lung Cancer	A confirmed clinical diagnosis of lung cancer documented within the patients past medical history (Yes or No)
Neuromuscular Disease	Any neurological or neuromuscular disease confirmed by clinical diagnosis documented within the patients past medical history such as strokes, epilepsy, and Parkinson disease (Yes or No)
Diabetes Mellitus	A confirmed clinical diagnosis of diabetes mellitus documented within the patients past medical history or a study participant who is prescribed long-term oral hypoglycaemic drugs or insulin (Yes or No)
Hypertension	A confirmed clinical diagnosis of hypertension documented within the patients past medical history or a study participant who is prescribed long-term antihypertensive drugs (Yes or No)
Ischaemic Heart Disease	A confirmed clinical diagnosis of ischaemic heart disease documented within the patients past medical history or a study participant who is prescribed a long-term antiplatelet drug (Yes or No)

Independent Predictor Variables (p=44)	Definition
Chronic Kidney Disease	A confirmed clinical diagnosis of chronic kidney disease documented within the patients past medical history or a baseline estimated glomerular filtration rate of < 60 (Yes or No)
Non-steroidal anti-inflammatory drugs	Repeat prescription, within the last 3 months, of oral non-steroidal anti-inflammatory drugs documented within the patient's drug history (Yes or No)
Anticoagulant	Repeat prescription, within the last 3 months, of anticoagulation documented within the patient's drug history (Yes or No)
Antibiotics	Repeat prescription, within the last 3 months, of antibiotics documented within the patient's drug history (Yes or No)
Immunosuppressants	Repeat prescription, within the last 3 months, of immunosuppressants, excluding corticosteroids and previous completed chemotherapy, documented within the patient's drug history (Yes or No)
Corticosteroids	Repeat prescription, within the last 3 months, of oral corticosteroids documented within the patient's drug history (Yes or No)
Angiotensin Converting Enzyme Inhibitors	Repeat prescription, within the last 3 months, of angiotensin converting enzyme inhibitors documented within the patient's drug history (Yes or No)
Angiotensin Receptor Blockers	Repeat prescription, within the last 3 months, of angiotensin receptor blockers documented within the patient's drug history (Yes or No)
CT imaging severity of COVID-19 related changes	Extent of air-space consolidation or ground-glass opacification in lung tissue on CT scans, where available, performed during inpatient stay (unilateral or bilateral)
COVID-19 related Chest X-Ray changes	Shadowing, consolidation, or haziness attributed to COVID-19 by a consultant radiologist on a chest X-Ray, where available, performed during inpatient stay (Yes or No)
Lactate	Lactate level displayed on blood gas measurement, where available, performed on the day of COVID-19 clinical presentation (mmol/L)
Lymphocytes	Lymphocyte count in blood sample obtained by venepuncture, where available, performed on the day of COVID-19 clinical presentation (10^9/L)
Neutrophils	Neutrophil count in blood sample obtained by venepuncture, where available, performed on the day of COVID-19 clinical presentation (10^9/L)
Albumin	Minimum albumin across all blood samples obtained by venepuncture, where available, throughout the inpatient stay (g/L)
Ferritin	Maximum ferritin across all blood samples obtained by venepuncture, where available, throughout the inpatient stay (ng/L)
D-Dimer	Maximum D-Dimer across all blood samples obtained by venepuncture, where available, throughout the inpatient stay (ng/ml)
Fibrinogen	Maximum fibrinogen across all blood samples obtained by venepuncture, where available, throughout the inpatient stay (g/L)
Troponin	Maximum troponin across all blood samples obtained by venepuncture, where available, throughout the inpatient stay (local units)
C-Reactive Protein (CRP) Day 0	CRP in blood sample obtained by venepuncture, where available, performed on the day of COVID-19 clinical presentation (mg/L)
CRP Day 1-2	Maximum CRP in blood sample obtained by venepuncture performed between days 1 and 2 since the day of COVID-19 clinical presentation, where available (mg/L)

Independent Predictor Variables (p=44)	Definition
CRP Day 3-4	Maximum CRP in blood sample obtained by venepuncture performed between days 3 and 4 since the day of COVID-19 clinical presentation, where available (mg/L)
CRP Day 5-6	Maximum CRP in blood sample obtained by venepuncture performed between days 5 and 6 since the day of COVID-19 clinical presentation, where available (mg/L)
CRP Day 7-8	Maximum CRP in blood sample obtained by venepuncture performed between days 7 and 8 since the day of COVID-19 clinical presentation, where available (mg/L)
CRP Day 9-10	Maximum CRP in blood sample obtained by venepuncture performed between days 9 and 10 since the day of COVID-19 clinical presentation, where available (mg/L)
CRP Day 11-12	Maximum CRP in blood sample obtained by venepuncture performed between days 11 and 12 since the day of COVID-19 clinical presentation, where available (mg/L)
CRP Day 13-14	Maximum CRP in blood sample obtained by venepuncture performed between days 13 and 14 since the day of COVID-19 clinical presentation, where available (mg/L)
CRP Day 15-20	Maximum CRP in blood sample obtained by venepuncture performed between days 15 and 20 since the day of COVID-19 clinical presentation, where available (mg/L)

Table S2: Patient Characteristics and descriptive statistics

**Demographics (n=355)**						
Male (%)	52					
Non-Caucasian (%)	30					
Age (Years)	Median	Upper Quartile	Lower Quartile	Interquartile Range	Mean	Standard Deviation
	65	81	52	29	65	18
**Vital Observations (n=355)**						
	Median	Upper Quartile	Lower Quartile	Interquartile Range	Mean	Standard Deviation
Oxygen Saturation (%)	93	96	89	7	91	8
Respiratory Rate (breaths per minute)	24	28	20	8	25	7
Temperature (°Celsius)	37.9	38.6	37.1	1.5	37.9	1.0
**Comorbidities and Medications (%) (n=355)**						

Obesity	34.3					
Previous Venous Thromboembolism	6.5					
Chronic Obstructive Pulmonary Disease	11.5					
Bronchiectasis	2					
Asthma	9.9					
Interstitial Lung Disease	2.3					
Lung Cancer	2.5					
Neuromuscular Disease	15.8					
Diabetes Mellitus	24.2					
Hypertension	36					
Ischaemic Heart Disease	12.1					
Chronic Kidney Disease	13.2					
Non-steroidal anti-inflammatory drugs	3.1					
Anticoagulant	16.9					
Antibiotics	3.1					
Immunosuppressants	5					
Corticosteroids	4.2					
Angiotensin Converting Enzyme Inhibitors	15.2					
Angiotensin Receptor Blockers	6.8					
**Radiographic Changes (%)**						
Bilateral COVID-19 CT changes (n=117)	91.5					
COVID-19 Chest X-Ray Changes (n=341)	69.5					
**Biochemical Markers**						
	Mean	Median	Upper Quartile	Lower Quartile	Interquartile Range	Standard Deviation
Lactate (mmol/L), n=242	1.85	1.4	2	1.1	0.9	1.63
Lymphocytes (10^9/L), n=351	1.10	1	1.3	0.7	0.6	0.67
Neutrophils (10^9/L), n=351	6.75	5.4	8.8	3.25	5.55	4.86
Albumin (g/L), n=341	31.38	31	36	27	9	6.6
Ferritin (ng/L), n=51	866.33	725	1371.5	264.5	1107	760.43
D-Dimer (ng/ml), n=115	2604.11	656	2163	344.5	1818.5	7016.26
Fibrinogen (g/L), n=18	44.91	5.85	8.56	4.41	4.15	121.97
Troponin (Local units), n=75	628.2	30.9	90.9	7.35	83.55	3201.81
C-Reactive Protein (CRP) Day 0 (mg/L), n=337	110.39	96	164	39	125	89.13
CRP Day 1-2 (mg/L), n=217	152.36	142	212	77	135	94.84
CRP Day 3-4 (mg/L), n=185	146	117	203	67	136	106.14

CRP Day 5-6 (mg/L), n=139	132.6	111	187	55.5	131.5	103.54
CRP Day 7-8 (mg/L), n=106	117.92	87.5	164	46	118	99.67
CRP Day 9-10 (mg/L), n=75	122.65	98	170.5	51.5	119	95.25
CRP Day 11-12 (mg/L), n=63	94.46	73	124.5	33.5	91	83.15
CRP Day 13-14 (mg/L), n=43	91.6	65	121	38.5	82.5	77.49
CRP Day 15-20 (mg/L), n=36	97.43	57.5	133.5	26.5	107	91.67

Table S3: The heat-mapped probabilities of inpatient death of patients at different age groups. As shown, the death rate of COVID-19 patients' ≥70 years is 5 times larger than patients' ≤40 years.

**Inpatient Mortality (IPD)**	**Age Category (Years)**		
	**≤ 40**	**41 – 69**	**70 ≤**
Yes	13.97%	31.50%	68.30%
No	86.03%	68.50%	31.70%

Table S4: The heat-mapped probabilities of death and survival of patients based on OS.

**Inpatient Mortality (IPD)**	**OS (%)**	
	**≥92**	**<92**
Yes	48.90%	50.90%
No	51.10%	49.10%

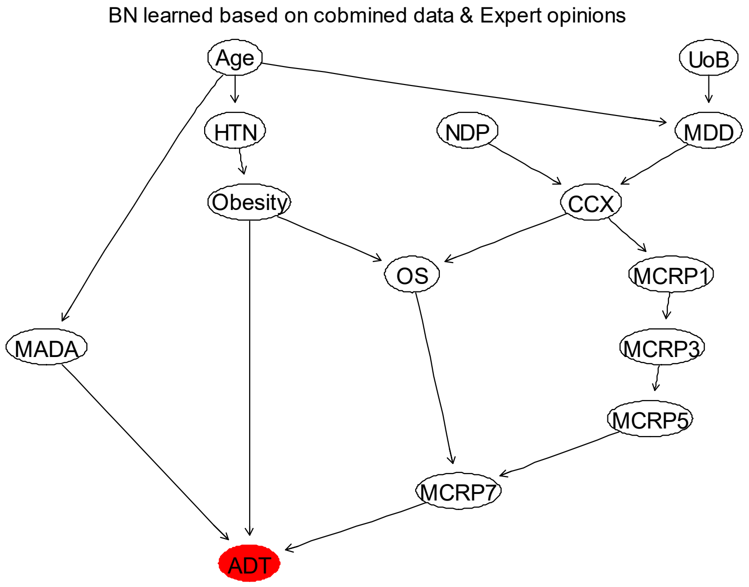

Figure S1: The BN that is learned from the combination of data and expert opinions, to model “ADT” in terms of the first 11 most relevant factors.

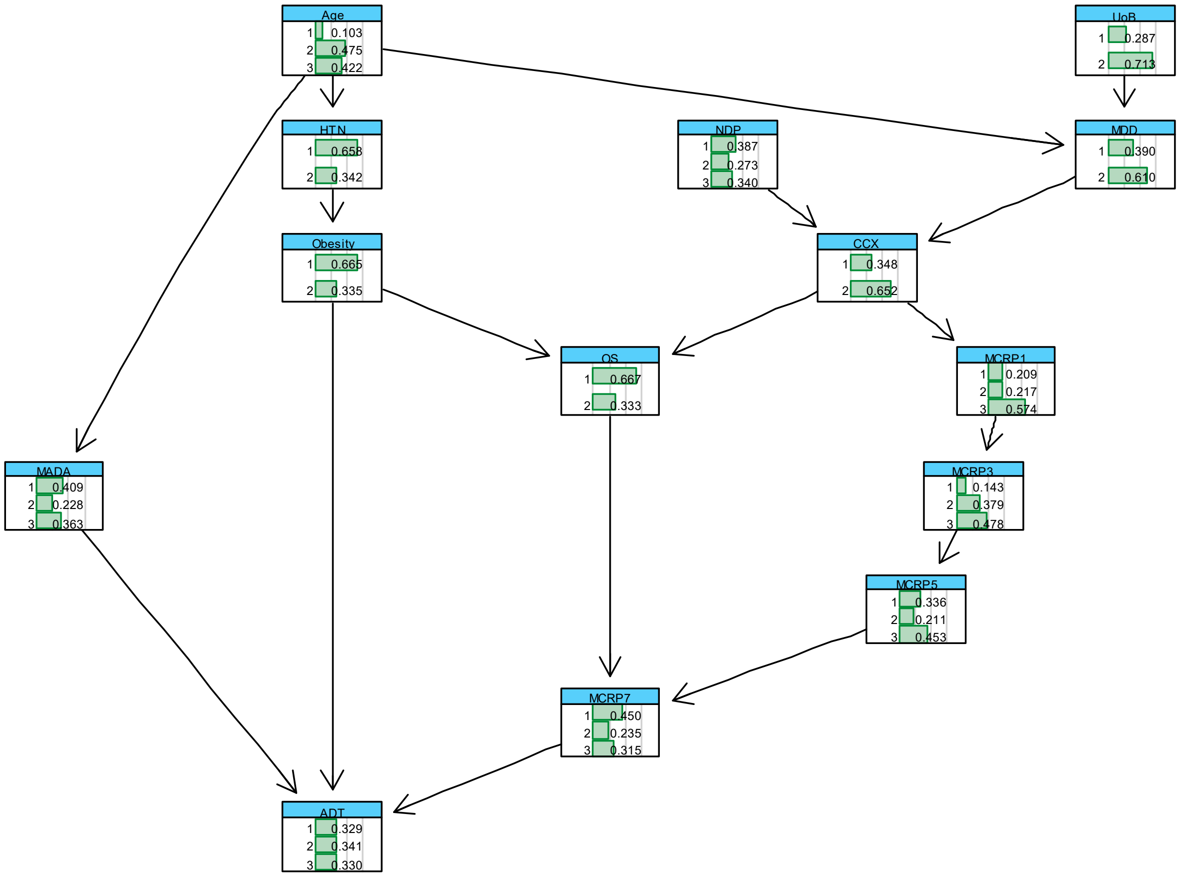

Figure S2: The BN with conditional probability tables learned for "ADT" outcome based on the combined elicited domain expert opinions with the (balanced) data.

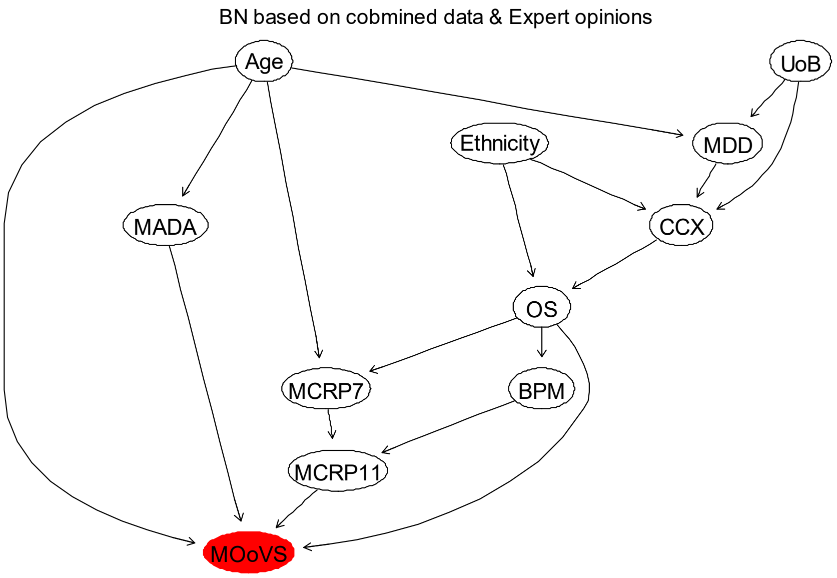

Figure S3: The BN that is learned from the combined data and non-domain expert opinions to model "MOoVS" in terms of the most influencing factors.

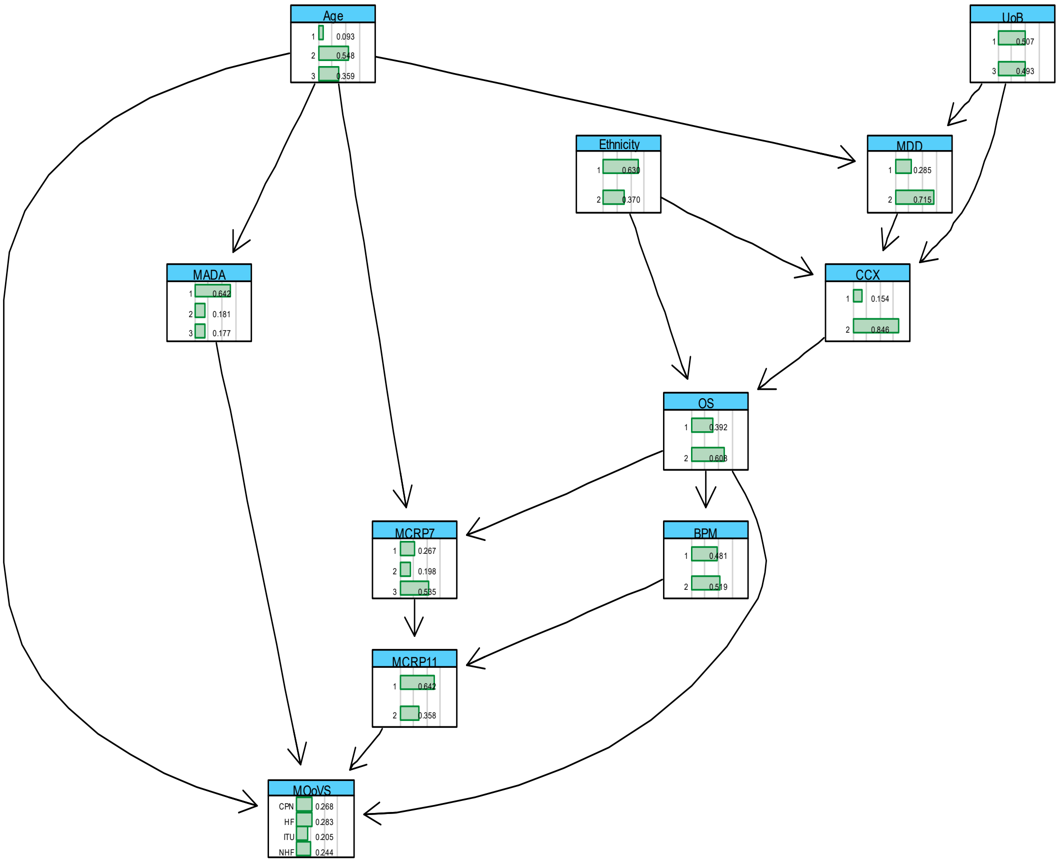

Figure S4: The BN with conditional probability tables learned for "MOoVS" outcome based on the combined elicited domain expert opinions with the (balanced) data.

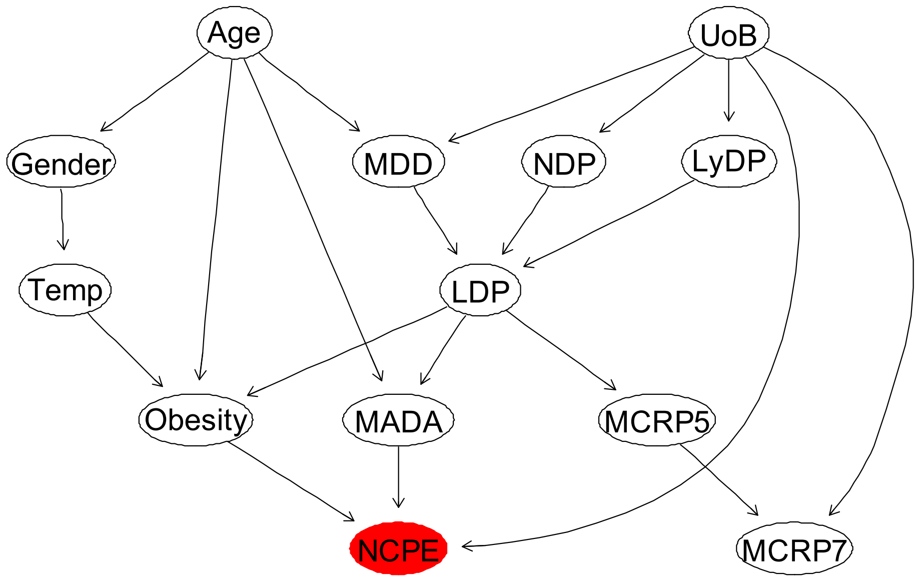

Figure S5: The BN that is learned from the combined data and domain expert opinions to model "NCPE" in terms of its most influencing factors.

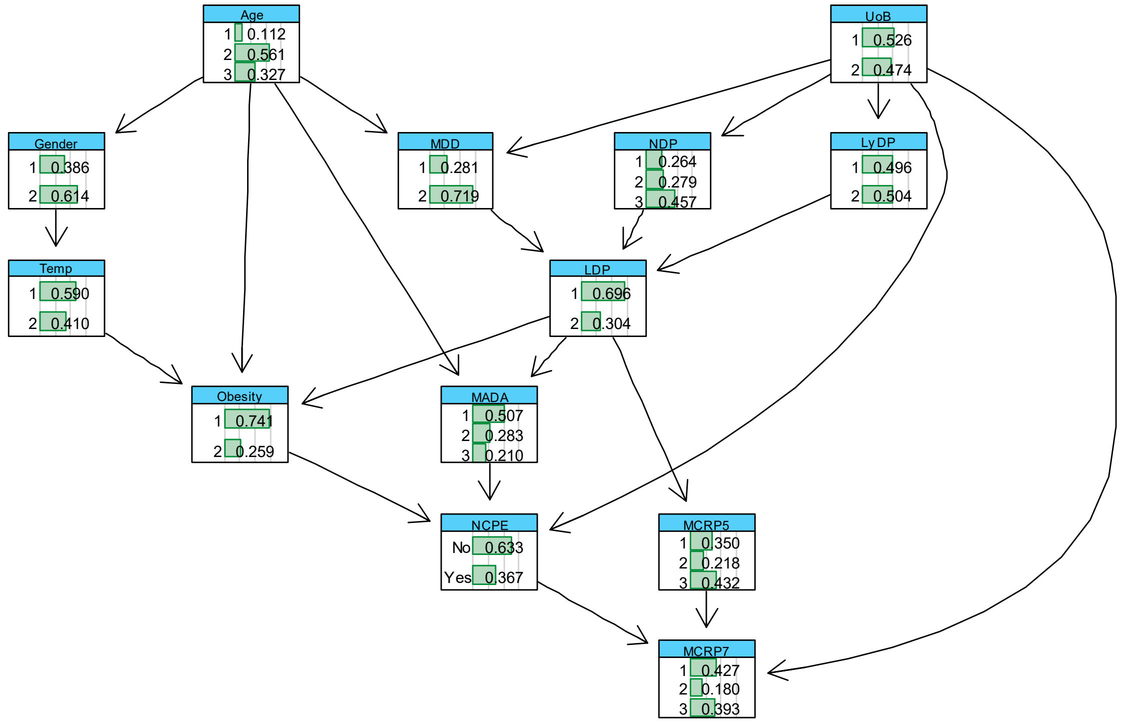

Figure S6: The BN for "NCPE", as shown in Supplementary Figure 5, augmented with the computed conditional probability tables learned from data. The marginal probability table of each variable, after the whole dataset was balanced with respect to the NCPE, can be observed on each node.
